# Economic burden of adult mild traumatic brain injury in the United States: a scoping review of healthcare-related charges and costs

**DOI:** 10.1016/j.lana.2026.101558

**Published:** 2026-07-06

**Authors:** Kathleen R. Ran, David J. Caldwell, Phiroz E. Tarapore, Cathra Halabi, Alexander A. Aabedi, Maria C.V. Velasco, Rajiv Saigal, Anthony M. DiGiorgio, Michael C. Huang, H.E. Hinson, Geoffrey T. Manley, John K. Yue

**Affiliations:** a Department of Neurosurgery, Johns Hopkins University School of Medicine, Baltimore, MD, USA; b Department of Neurological Surgery, University of California, San Francisco, San Francisco, CA, USA; c Brain and Spinal Injury Center, Zuckerberg San Francisco General Hospital, San Francisco, CA, USA; d Weill Institute for Neurosciences, University of California, San Francisco, San Francisco, CA, USA; e Department of Neurological Surgery, San Francisco Veterans Affairs Medical Center, San Francisco, CA, USA; f Department of Neurology, University of California, San Francisco, San Francisco, CA, USA; g Bakar Computational Health Sciences Institute, University of California, San Francisco, San Francisco, CA, USA; h Philip R. Lee Institute for Health Policy Studies, University of California, San Francisco, San Francisco, CA, USA

**Keywords:** Delivery of health care, Healthcare costs, Healthcare utilization, Hospital charges, Traumatic brain injury

## Abstract

Mild traumatic brain injury (mTBI) accounts for 80–90% of all traumatic brain injuries, yet its economic burden remains poorly characterized. We synthesized direct healthcare charges and costs associated with mTBI in the United States (U.S.). A PubMed search through October 14, 2025 identified primary studies reporting direct healthcare-related charges or costs among adults with mTBI. All estimates were standardized to 2025 U.S. dollars. Twenty-one studies (1996–2023) out of 3300 screened (0.64%) met inclusion criteria. Index hospitalization costs ranged from $3984 to $31,316 and remained stable over time, whereas national hospital charges increased from $26,021 (mean, standard deviation (SD) $1895) in 1996 to more than $172,080 in recent reports. First-year healthcare costs ranged from $16,898 to $29,045 in civilian populations and reached $182,094 (mean, SD $161,743) in military rehabilitation settings. These findings indicate mTBI imposes substantial costs beyond initial hospitalization and highlight growing financial pressures on trauma systems.

## Introduction

Mild traumatic brain injury (mTBI) accounts for 80–90% of all TBI with an estimated annual incidence exceeding two million adults in the United States (U.S.).^1^^,^^2^ Although direct mortality attributable to mTBI is low, the chronicity of post-injury symptoms can substantially impair daily functioning, employment, and quality of life for months to years in certain subpopulations.^2^^,^^3^ TBI comprised over 242,000 years lived with disability (YLDs) in the U.S. in 2021 estimates, of which mTBI contributed over 126,000 YLDs.^4^ These figures highlight the substantial population-level burden of mTBI-related disability.

Mild TBI is associated with recurrent emergency department visits, neuroimaging, and prolonged outpatient utilization that extend well beyond the index hospitalization.^5^^,^^6^ While substantial individual and societal healthcare costs are associated with mTBI, defining the precise components that contribute to its economic burden, including costs attributable to the index hospitalization, those accrued after hospitalization, and changes in charges compared to costs over time, remains elusive. Existing estimates vary widely across settings, payer types, and phases of care, reflecting methodological heterogeneity and the absence of standardized cost reporting.

Notably, the U.S. National Institutes of Health-National Institute of Neurological Disorders and Stroke (NIH-NINDS) published the 2025 Clinical, Biomarker, Imaging, and Modifier (CBI-M) Framework to advance the multidimensional classification and characterization of TBI. Implementation and validation of this framework is required for administrative datasets and economic studies.^7^ The term mTBI is thus used in this present review to align with terminology employed in the existing literature. Quantifying and contextualizing healthcare-related charges and costs are requisite to defining the scope of economic burden imposed by mTBI and informing value-based care strategies. Understanding how costs accumulate across acute and chronic phases of injury can inform resource allocation and bolster the development of multidisciplinary recovery pathways, while mitigating long-term expenditures. We conducted a scoping review of primary medical literature on U.S. mTBI-associated charges and costs, and standardized reported expenditure amounts to 2025 U.S. dollars (USD, $).

## Methods

### Search strategy and study selection

**Fig. 1:**
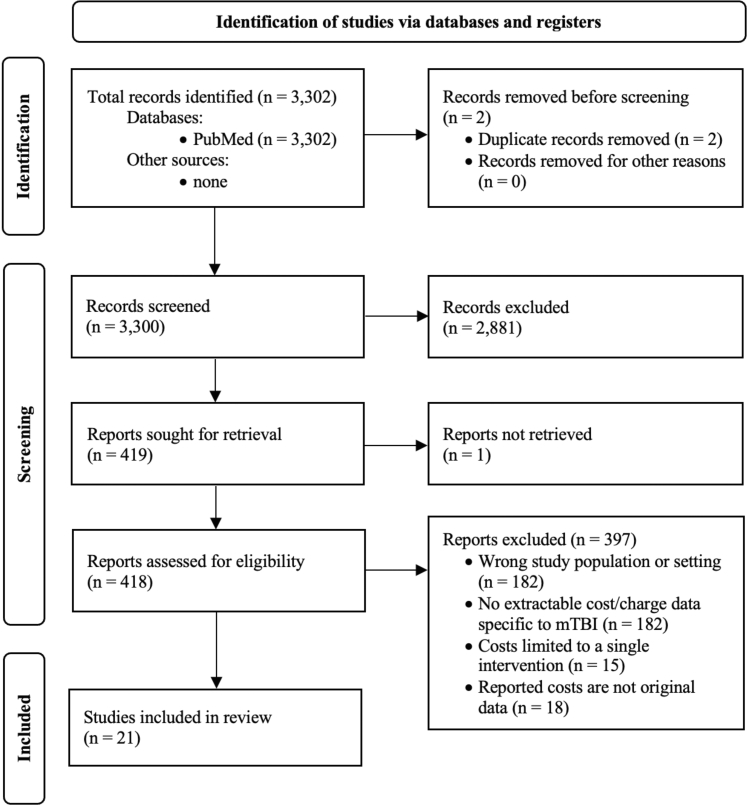
PRISMA flow diagram of study identification, screening, and inclusion. This diagram illustrates the PRISMA-based workflow used to identify, screen, and include studies in the scoping review. mTBI, mild traumatic brain injury.

This scoping review was conducted in accordance with the Preferred Reporting Items for Systematic Reviews and Meta-analyses (PRISMA) extension for Scoping Reviews reporting guidelines (Fig. 1). Prespecified search criteria were developed, and the National Library of Medicine PubMed database was queried from inception to October 14, 2025, using keywords and Medical Subject Heading (MeSH) terms relevant to the economic burden of TBI. The full search string, including all MeSH terms and Boolean operators, is provided in Supplementary Table S1. Inclusion criteria were primary published reports on adult patients (≥18 years) with clinically-diagnosed mTBI as specified in each report, with original, quantitative cost and/or charge data from U.S. settings. Mild TBI was defined according to the criteria used in each included study. Definitions encompassed Glasgow Coma Scale (GCS) scores of 13–15, Head Abbreviated Injury Scale (AIS) scores of 1–2, American Congress of Rehabilitation Medicine (ACRM) criteria, 2 + 10 TBI Screening Questionnaire and Military Acute Concussion Evaluation (MACE), International Classification of Diseases (ICD-9/10) diagnostic codes, Department of Defense (DoD) and Veterans Affairs (VA) mTBI diagnostic criteria, Brain Injury Guideline (BIG) and modified BIG (mBIG) scores, as well as additional findings on clinical exam and neuroimaging. Studies that did not separate data reporting based on TBI severity were excluded.

### Data extraction and quality assessment

For each report included in this scoping review, K.R.R. extracted data pertaining to the following variables: study population; study period; setting; mTBI definition; cost type; phase of care evaluated; and methods of cost estimation. K.R.R. evaluated the methodological quality of included studies using the Newcastle–Ottawa Scale (NOS), a validated instrument for observational studies. The NOS evaluates three domains: selection, comparability, and ascertainment of outcome or exposure (maximum 9 stars). Studies scoring 7–9 stars were classified as high quality, 4–6 as moderate quality, and <4 as low quality. The completeness of economic reporting in each study was separately characterized using a Consolidated Health Economic Evaluation Reporting Standards (CHEERS)-based framework encompassing 10 reporting domains: title and abstract clarity; background and objectives; study population definition; setting and location; time horizon; cost type; costing methodology; price year and currency reporting; and characterization of heterogeneity and uncertainty. In addition, studies were assigned a level of evidence according to the Oxford Centre for Evidence-Based Medicine (OCEBM) 1–5 scale. Final study selections were reviewed by D.J.C and approved by the senior author (J.K.Y.).

### Healthcare cost classification and comparison

Outcome data were extracted by K.R.R. The primary outcomes were direct healthcare-related charges and costs associated with mTBI. Outcomes were further stratified by population type (civilian versus military/VA). Costs were categorized by phase of care: acute (index emergency or inpatient episode), post-acute (rehabilitation, outpatient care, and additional hospital encounters within the first year of injury), and long-term (>1 year post-injury), and by cost type (healthcare charges versus cost). Healthcare costs were defined as the actual economic resources consumed in delivering care, whereas healthcare charges referred to the amounts billed by hospitals or providers. Where feasible, total longitudinal costs were decomposed into relative proportions of acute, post-acute, and chronic expenditures. Costs were converted to 2025 USD using the Consumer Price Index for All Urban Consumers.^8^

### Statistical analysis

Charges and costs were summarized using descriptive statistics. For studies reporting mean costs for study population subgroups, weighted means were calculated. Median values for subgroups were reported separately. Primary comparisons were: (1) healthcare charges versus costs for equivalent populations and time periods; (2) costs across phases of care (acute, post-acute, long-term); and (3) costs across civilian versus military and VA populations.

### Ethics

No ethical approval was required, as this was a scoping review paper that did not involve identifiable or individual patient data.

### Role of the funding source

The funders of the study had no role in study design, data collection, data analysis, data interpretation, writing of the report, or the decision to submit the paper for publication.

## Results

**Fig. 2:**
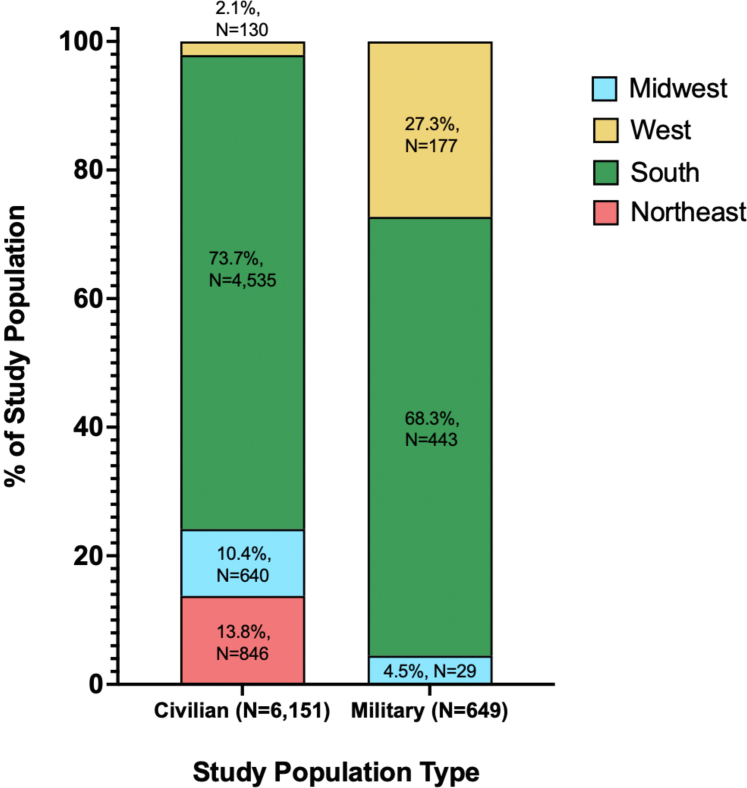
Geographic distribution of civilian and military study populations. This figure illustrates the proportional geographic distribution of included civilian (N = 6151) and military (N = 649) study populations, excluding studies that used national databases without site-level geographic information. Civilian and military cohorts were subdivided by U.S. Census region. For studies by Richardson et al. (2018) and Dismuke-Greer et al. (2023), site-specific population sizes were not reported; therefore, total cohort sizes were evenly divided across listed sites to estimate the contribution of each region.

Twenty-one U.S. studies out of 3300 screened (0.64%) met inclusion criteria (Fig. 1, Table 1). Study periods spanned 1996–2023. Study populations ranged from single-center cohorts to national civilian and military repositories, including four (19%) unique general population-based databases and two (9.5%) unique military databases. Among seventeen studies with identifiable sites, nine (53%) civilian and eight (47%) military or VA centers were represented (Supplementary Table S2). Across both civilian and military populations, the greatest proportion of patients were from Southern U.S. Study populations, settings, and geographic distributions are summarized in Fig. 2 and Supplementary Table S2.

**Table 1:** Summary of included studies reporting mild TBI-associated costs and/or charges.

Publication	Setting	Study year(s)	N	Mild TBI definition	Study objectives	Quantified cost types
Schootman et al., 2003	National database: Nationwide Inpatient Sample	1996	2384	AIS 1-2	To investigate hospitalization charges for TBI	Inpatient healthcare charges
Farhad et al., 2013	National database: Nationwide Inpatient Sample	2006–2007	48,708	AIS 1-2	To compare TBI hospitalization outcomes between two time periods, 1993–1994 and 2006–2007	Inpatient healthcare charges
Salisbury et al., 2017	Level 1 trauma center; United States Southwest	2006–2014	2855	LOC <1 h	To investigate hospitalization charges for TBI	Inpatient healthcare charges
Taylor et al., 2017	Department of Veterans Affairs Department of Defense administrative data sets	2010–2013	7318	American Congress of Rehabilitation Medicine Criteria for mTBI	To assess Veterans Health Administration healthcare utilization and costs for veterans with TBI	Healthcare costs three years post-TBI
Martyak et al., 2018	Level 1 trauma center in Virginia	2014–2017	143	GCS 13–15 and ICH on CT	To assess the effect of the implementation of the mild TBI protocol on resource utilization and patient safety	Itemized charges of head CT and neurosurgery consult
Richardson et al., 2018	TBI clinics of two military medical centers (Joint Base Lewis McChord, Washington and Fort Bragg, North Carolina)	2011–2013 (unclear study end date)	356	2 + 10 Traumatic Brain Injury Screening Questionnaire and Military Acute Concussion Evaluation	To compare outcomes after problem solving therapy versus education only therapy for combat-related TBI	Rehabilitation treatment costs
Pavlov et al., 2019	National database: Optum claims	2006–2016	80,004	ICD-9-CM codes	To study healthcare resource utilization and costs in the 12-month period post-TBI	Healthcare costs one-year post-TBI
Dengler et al., 2020	Tertiary care center in Texas	2004–2013	1447	GCS 14–15 and CT with 1) depressed skull fracture, and/or 2) a trauma-related intracranial abnormality	To determine the rate of secondary overtriage and associated costs for complicated mild TBI	Inpatient healthcare charges
Dismuke-Greer et al., 2020	Four large Veterans Affairs Medical Centers: (Richmond, Virginia; Tampa, Florida; Minneapolis, Minnesota; Palo Alto, California)	2002–2016	400	DoD criteria for mTBI	To compare health services utilization and costs in veterans with blast-related mild TBI versus non-blast-related mild TBI	Annual outpatient healthcare costs during entire study period
Root et al., 2020	Level 1 trauma center in New Hampshire	2015–2018 for intervention arm, 2012–2015 for control arm	145 (N = 60 intervention, N = 85 control)	Normal neurological exam (except transient alteration in consciousness) and CT with 1 or more of the following 3 injuries: (1) cerebral contusions <1 cm in maximum extent, (2) convexity subarachnoid hemorrhage, or (3) closed, non-displaced skull fractures	To test the cost effectiveness of managing mild TBI in an emergency department observation unit.	Inpatient healthcare charges
Cogan et al., 2022	National database: IQVIA Integrated Data Warehouse	2011–2016	40,882	ICD-9 or ICD-10 code and either (1) outpatient diagnosis of TBI within the professional fee claims database or (2) an ED-based TBI diagnosis without evidence of an inpatient stay within 1 day of the diagnosis	To compare the economic burden of health care resource utilization among adults with and without chronic vestibular impairment after mTBI	Healthcare costs at one and two years post-TBI
Dalton et al., 2022	Military Health System Data Repository	2007–2011	382	AIS1-2	To evaluate healthcare expenditures among United States military service members with TBI	Healthcare costs over several years post-TBI
Dismuke-Greer et al., 2023	Five rehabilitation centers in the Veterans Health Administration (Richmond, Virginia; Tampa, Florida; Minneapolis, Minnesota; Palo Alto, California; San Antonio, Texas)	2010–2020	143	Post-traumatic amnesia <24 h	To examine first year hospitalization costs for TBI based on severity stratified by post-traumatic amnesia	Healthcare costs at one-year post-TBI
Harris et al., 2024	Level 1 trauma center in Ohio	2015–2018	596	GCS 13-15	To determine which of the published definitions for mTBI performs best in the acute care setting at identifying mild TBI patients, including the potential cost and resource savings	Itemized costs of head CT and ICU stay
Ranson et al., 2024	Urban academic medical center in New York	2014–2021	701	AIS 1-2	To characterize the effects of head injuries on hospital quality measures, costs, and outcomes in patients with concomitant orthopedic trauma	Inpatient healthcare costs
Shen et al., 2024	Level 1 trauma center in California	2017–2022	130	mBIG1, mBIG2	To assess safety and potential resource savings associated with the application of mBIG on interhospital patient transfers for TBI	Inpatient healthcare charges
Beard et al., 2025	Level 1 trauma center in rural Midwest	2020–2022	44	BIG1	To compare outcomes and healthcare expenditures of BIG1 TBI patients transferred from rural critical-access hospitals to those directly admitted to the trauma center	Inpatient healthcare costs
Darr et al., 2025	Large military treatment facility in Southern California	Not specified	148	DoD criteria for mTBI	To compare two cognitive rehabilitation approaches in active-duty service members with a history of mTBI and post-concussive syndrome	Rehabilitation treatment costs
Dobbs et al., 2025	Level 1 trauma center in southern West Virginia	2021–2023	90	mBIG1 and mBIG2	To evaluates how adopting the mBIG criteria affects outcomes and resource utilization for TBI	Inpatient healthcare costs
Marcet et al., 2025	Merative MarketScan Research Database	2000–2022	105,324	ICD-9 or ICD-10 codes	To compare health care utilization and costs associated with TBI and SCI versus isolated TBI	Index hospitalization and one-year post-TBI healthcare costs
Richard et al., 2025	Military Data Repository	2011–2021	51,009	mTBI based on provider diagnosis but criteria not specified	To study differences in costs, utilization, and quality of care provided by primary care physicians versus nurse practitioners and physician assistants for mTBI	Primary care costs for study period

**Caption:** This table summarizes all included U.S.-based studies that quantified direct healthcare costs or charges related to mild TBI. Extracted variables include study setting, study years, study population size, definition of mild TBI, primary study objectives, and the cost types reported.

AIS, Abbreviated Injury Scale; BIG, brain injury guideline; CT, computed tomography; DoD, Department of Defense; ED, emergency department; GCS, Glasgow Coma Scale; ICD, International Classification of Diseases; ICD-10, International Classification of Diseases, Tenth Edition; ICD-9, International Classification of Diseases, Ninth Edition; ICD-9-CM, International Classification of Diseases, Ninth Edition, Clinical Modification; ICH, intracranial hemorrhage; ICU, intensive care unit; IQVIA, Intercontinental Marketing Statistics, Quintiles, Via (company); LOC, loss of consciousness; mBIG, modified brain injury guideline; mTBI, mild traumatic brain injury; SCI, spinal cord injury; TBI, traumatic brain injury.

Definitions of mild TBI varied (Table 1). Head AIS score of 1–2 was the most common standalone criterion used for defining mTBI. Other definitions incorporated the ICD-9/10 diagnostic codes, GCS score 13–15, radiographic findings on neuroimaging, clinical symptoms scales, and BIG or mBIG scores of 1–2. Three out of the twenty-one (14%) studies applied military-specific mTBI diagnostic criteria. Quality assessment using the NOS identified twenty studies as high quality (7–9 stars) and one study (Pavlov et al., 2019) as moderate quality (6 stars); no studies were rated low quality (Supplementary Table S3). The most common sources of methodological limitation were absence of a true non-exposed comparator group and reliance on cross-sectional administrative data designs. Studies were further designated as high quality if they fulfilled all CHEERS reporting domains. Those that did not fully satisfy one or more domains were classified as moderate quality. Of the included studies, six (29%) were classified as high quality and fifteen (71%) as moderate quality (Supplementary Table S4). All included studies satisfied at least 8 of the 10 reporting domains. High-quality studies predominantly captured costs within the first-year post-injury and beyond, while moderate-quality studies more commonly reported costs limited to the index hospitalization encounter.

### Index hospitalization costs of mTBI

**Fig. 3:**
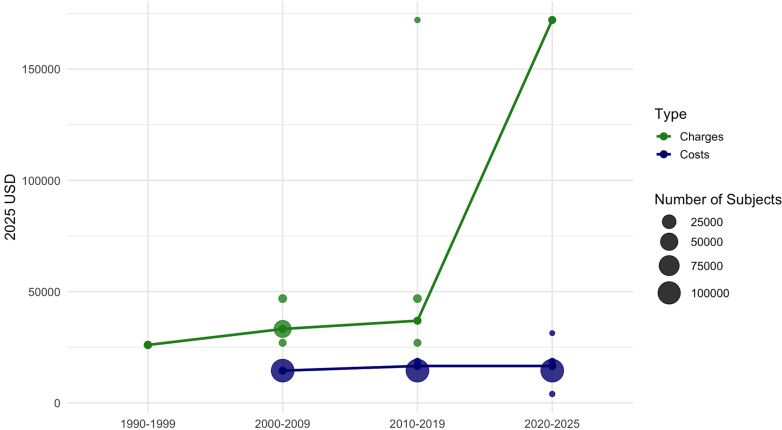
Temporal trends in healthcare costs and charges for acute inpatient care for mild traumatic brain injury. This figure displays reported healthcare costs and charges associated with index hospitalization for mild TBI, grouped by study period. Each point represents an individual study population, with point size proportional to sample size. Costs and charges are plotted separately, and connecting lines represent median values. All values are standardized to 2025 U.S. dollars. TBI, traumatic brain injury; USD, United States dollars.

Ten out of twenty-one (48%) studies quantified inpatient expenditures, including four (19%) that reported healthcare costs and six (29%) that reported healthcare charges (Table 2).^9^, ^10^, ^11^, ^12^, ^13^, ^14^, ^15^, ^16^, ^17^, ^18^ All of these were civilian studies. For analytic clarity, findings are summarized by expenditure type (Fig. 3). Reported hospitalization costs ranged from $3984 to $31,316 (2025 USD) across institutions and health systems. Healthcare cost of the index hospitalization measured within the Merative MarketScan database was $14,474 (median, interquartile range (IQR) not reported, median absolute deviation (MAD) $7777) during 2000–2022.^11^

**Table 2:** Total inpatient hospitalization costs and/or charges associated with index hospitalization for mild TBI.

Publication	Setting	Study year(s)	Costs/Charges	Method of cost/Charge estimation	Reported costs/Charges	2025 USD equivalent of costs/Charges
Ranson et al., 2024	Urban academic medical center in New York	2014–2021	Healthcare costs	Hospital finance department	$16,214 (mean, SD $20,631), AIS 1, N = 511 $13,010 (mean, SD $13,333), AIS 2, N = 190 $15,346 (weighted mean, SD $18,977), N = 701 2021 USD^a^	$19,691 (mean, SD $25,055), AIS 1, N = 511 $15,800 (mean, SD $16,192), AIS 2, N = 190 $18,637 (weighted mean, SD $23,046), N = 701 2025 USD
Beard et al., 2025	Level 1 trauma center in rural Midwest	2020–2022	Healthcare costs	Value of CPT codes for healthcare services obtained from FAIR Health Consumer Database	$23,005 (mean, SD not reported); $20,175 (median, IQR not reported), direct admissions, N = 28 $33,997 (mean, SD not reported); $29,914 (median, IQR not reported), transfers, N = 21 $27,715.86 (weighted mean, SD not reported), N = 49 2022 USD^a^	$25,994 (mean, SD not reported); $22,796 (median, IQR not reported), direct admissions, N = 28 $38,413 (mean, SD not reported); $33,800 (median, IQR not reported), transfers, N = 21 $31,316 (weighted mean, SD not reported), N = 49 2025 USD
Marcet et al., 2025	Merative MarketScan Research Database	2000–2022	Healthcare costs	Merative MarketScan Research Database	$12,810 (median, IQR not reported, MAD $6883) 2022 USD	$14,474 (median, IQR not reported, MAD $7777) 2025 USD
Dobbs et al., 2025	Level 1 trauma center in southern West Virginia	2021–2023	Healthcare costs	Institution's Accounts Department	$3751.53 (mean, SD not reported) 2023 USD^a^	$3984 (mean, SD not reported) 2025 USD^a^
Schootman et al., 2003	NIS	1996	Healthcare charges	Billed charges reported in the NIS database	$12,647 (mean, SD $921); $6061 (median, IQR not reported) 1996 USD^a^	$26,021 (mean, SD $1895); $12,470 (median, IQR not reported) 2025 USD
Farhad et al., 2013	NIS	2006–2007	Healthcare charges	Healthcare charges reported in the NIS database	$21,160 (mean, SD $20,500) 2007 USD^a^	$33,208 (mean, SD $32,173) 2025 USD
Salisbury et al., 2017	Level 1 trauma center in United States Southwest	2006–2014	Healthcare charges	Billed charges reported in the Dallas-Fort Worth Hospital Council Foundation utilization database	$34,500 (median, IQR $22,200–$61,800) 2014 USD^a^	$46,853 (median, IQR $30,149–$83,928) 2025 USD
Dengler et al., 2020	Tertiary care center in Texas	2004–2013	Healthcare charges	Institutional trauma registry data	$20,422.2 (median, IQR $14,833.20–$27,731.60), primary admissions, N = 780 $16,313.0 (median, IQR $1164.0–$24,316.9), secondary admissions by ambulance, N = 489 $19,555.8 (median, IQR $11,953.1–$28,301.9), secondary admissions by helicopter, N = 178 2013 USD^a^	$28,172 (median, IQR $20,462–$38,256), primary admissions, N = 780 $22,504 (median, IQR $1606–$33,545), secondary admissions by ambulance, N = 489 $26,977 (median, IQR $16,489–$39,042), secondary admissions by helicopter, N = 178 2025 USD
Root et al., 2020	Level 1 trauma center in New Hampshire	2015–2018 for intervention arm, 2012–2015 for control arm	Healthcare charges	Dartmouth–Hitchcock Medical Center patient records	$11,430.25 (mean, SD $6278.84), ED observation unit, N = 60 $14,858.76 (mean, SD $8536.01), admitted to hospital, N = 85 $13,447.65 (weighted mean, SD $7844), N = 145 2018 USD^a^	$14,649 (mean, SD $8047), ED observation unit, N = 60 $19,043 (mean, SD $10,940), admitted to hospital, N = 85 $17,235 (weighted mean, SD $10,053), N = 145 2025 USD
Shen et al., 2024	Level 1 trauma center in California	2017–2022	Healthcare charges	Institutional trauma registry data	$152,296 (median, IQR not reported), mBIG1, N = 69 $149,550 (median, IQR not reported), mBIG2, N = 61 2022 USD^a^	$172,080 (median, IQR not reported), mBIG1, N = 69 $168,978 (median, IQR not reported), mBIG2, N = 61 2025 USD

**Caption:** This table summarizes all included studies reporting total inpatient costs or hospital charges associated with the index mild TBI hospitalization. Extracted variables include study setting, study years, cost or charge type, method of cost or charge estimation, original reported values, and the corresponding 2025 USD equivalents following inflation adjustment.

Therefore, the reference year for the reported USD values was assumed to correspond to the final year of the study period.

AIS, Abbreviated Injury Scale; BIG, brain injury guideline; CPT, Current Procedural Terminology; ICU, intensive care unit; IQR, interquartile range; MAD, median absolute deviation; mBIG, modified brain injury guideline; mTBI, mild traumatic brain injury; NIS, Nationwide Inpatient Sample; obs, observation; SD, standard deviation; TBI, traumatic brain injury; USD, United States dollars.

a The study did not specify the reference year for reported costs or charges.

Compared to healthcare costs, billed charges were generally much higher. The earliest estimate, derived from the 1996 Nationwide Inpatient Sample (NIS), reported an average charge of $26,021 (mean, standard deviation (SD) $1895).^13^ By 2006–2007, NIS data showed charges rising to $33,208 (mean, SD $32,173).^14^ Charges continued to increase through the 2010s, with considerable variability across centers. The estimate by Shen et al. ($172,080) markedly exceeded consumer price index-adjusted trends from other institutions; however, the overall trajectory of escalating inpatient charges over time remained consistent, even when excluding this extreme value.^18^

### Healthcare costs within the first year after mTBI

Six out of twenty-one (29%) studies reported healthcare costs within the first year following mTBI (Table 3).^11^^,^^19^, ^20^, ^21^, ^22^, ^23^ National general population-based databases (Optum, MarketScan, and Intercontinental Marketing Statistics, Quintiles, Via (IQVIA)) estimated first-year costs between $16,898 (median, IQR not reported, MAD $5702) and $29,045 (mean, SD not reported). In contrast, national military and VA datasets reported costs from $16,616 (mean, SD not reported) to $94,413 (median, IQR $52,562–$191,776). The highest costs were observed in VA rehabilitation centers, which reported a one-year expenditure of $182,094 (mean, SD $161,743).

**Table 3:** Healthcare costs and/or charges in the first-year post-injury.

Publication	Setting	Study year(s)	Cost/Charge description	Method of cost/Charge estimation	Reported costs/Charges	2025 USD equivalent of cost/Charges
Taylor et al., 2017	Department of Veterans Affairs Department of Defense administrative data sets	2010–2013	Healthcare costs	VA Managerial Cost Accounting National Cost Extracts	$12,045 (mean, SD not reported); $7418 (median, IQR $4142–$13,428) 2013 USD^a^	$16,616 (mean, SD not reported); $10,233 (median, IQR $5714–$18,524) 2025 USD
Pavlov et al., 2019	Optum database	2006–2016	Healthcare costs. Included all medical and pharmacy costs.	Optum database	$9369 (mean, SD $36,051), 18–25 yrs, N = 12,142 $19,661 (mean, SD $50,971), 26–64 yrs, N = 23,588 $38,380 (mean, SD $69,092), 65+ yrs, N = 8348 $20,376 (weighted mean, SD $52,415), N = 44,078 2016 USD	$12,563 (mean, SD $48,339), 18–25 yrs, N = 12,142 $26,363 (mean, SD $68,345), 26–64 yrs, N = 23,588 $51,462 (mean, SD $92,643), 65+ yrs, N = 8348 $27,321 (weighted mean, SD $70,281), N = 44,078 2025 USD
Cogan et al., 2022	IQVIA Integrated Data Warehouse	2011–2016	Healthcare costs. Included pharmacy, outpatient, and inpatient visits.	IQVIA Integrated Data Warehouse	$27,664 (mean, SD not reported), mTBI + CVI, N = 20,441 $16,742 (mean, SD not reported), mTBI only, N = 20,441 $22,203 (weighted mean, SD not reported), N = 40,882 2017 USD	$36,189 (mean, SD not reported), mTBI + CVI, N = 20,441 $21,901 (mean, SD not reported), mTBI only, N = 20,441 $29,045 (weighted mean, SD not reported), N = 40,882 2025 USD
Dalton et al., 2022	Military Health System Data Repository	2007–2011	Healthcare costs. Included encounters at military facilities, civilian facilities, and pharmacies.	Military Health System Data Repository	$74,810 (median, IQR $41,648–$151,957) 2019 USD	$94,413 (median, IQR $52,562–$191,776) 2025 USD
Dismuke-Greer et al., 2023	Five rehabilitation centers in the Veterans Health Administration	2010–2020	Healthcare costs	VHA hospital costs were estimated from the Health Economics Resource Center discharge data sets based on Managerial Cost Accounting data available in VINCI	$149,943 (mean, SD $133,185) 2021 USD	$182,094 (mean, SD $161,743) 2025 USD
Marcet et al., 2025	Merative MarketScan Research Database	2000–2022	Healthcare costs	Merative MarketScan Research Database	$14,955 (median, IQR not reported, MAD $5046), 12-mo post-discharge costs $42,050 (median, IQR not reported, MAD $8843), index hospitalization + 12-mo post-discharge costs 2022 USD	$16,898 (median, IQR not reported, MAD $5702), 12-mo post-discharge costs $47,513 (median, IQR not reported, MAD $9992), index hospitalization + 12-mo post-discharge costs 2025 USD

**Caption:** This table summarizes all included studies reporting healthcare costs incurred during the first year following mild TBI. Extracted variables include study setting, study years, description of the cost or charge categories, method of cost or charge estimation, original reported values, and corresponding 2025 USD equivalents following inflation adjustment.

Therefore, the reference year for the reported USD values was assumed to correspond to the final year of the study period.

BIG, brain injury guideline; CPT, Current Procedural Terminology; CVI, chronic vestibular impairment; ICU, intensive care unit; IQR, interquartile range; MAD, median absolute deviation; mBIG, modified brain injury guideline; N, sample size; pts, patients; SD, standard deviation; TBI, traumatic brain injury; USD, United States dollars; VA, Veterans Affairs; VHA, Veterans Health Affairs; VINCI, Veterans Affairs Informatics and Computing Infrastructure; yrs, years.

a The study did not specify the reference year for reported costs or charges.

### Long-term healthcare costs after mTBI

Five out of twenty-one (24%) studies reported healthcare costs associated with mTBI beyond the first year post-injury (Table 4).^19^^,^^21^^,^^22^^,^^24^^,^^25^ Contemporary civilian claims data from IQVIA reported $48,561 (mean, SD $82,501) in cumulative costs during the first two years after mild TBI, highlighting substantial post-acute expenditures.^21^ Similarly, within the VA and DoD administrative data sets, annualized costs remained persistently elevated beyond the first year post-injury, comprising $9535 (mean, SD not reported) in the second year and $8637 (mean, SD not reported) in the third year.^19^ Concordantly, average annual healthcare costs reported by VA medical centers were $7332 (mean, 95% confidence interval (CI) $6853–$7979), and extended follow-up costs in the comprehensive Military Health System dataset were $14,223 (median, IQR $5019–$30,721).^22^^,^^24^ Encounter-level analyses of primary care utilization in military treatment facilities reported mean costs of $651 (mean, 95% CI $637–$666) per outpatient visit.^25^

**Table 4:** Healthcare costs and/or charges beyond the first-year post-injury.

Publication	Setting	Study year(s)	Cost/Charge description	Method of cost/Charge estimation	Reported costs/Charges	2025 USD equivalent of cost/Charges
Taylor et al., 2017	Department of Veterans Affairs Department of Defense administrative data sets	2010–2013	Healthcare costs. Total costs (includes inpatient, outpatient, pharmacy, and fee basis) during 2nd and 3rd year post-injury.	VA Managerial Cost Accounting National Cost Extracts	$6912 (mean, SD not reported); $2423 (median, IQR $505–$6857) $6261 (mean, SD not reported); $1976 (median, IQR $279–$5971) 2013 USD^a^	$9535 (mean, SD not reported); $3343 (median, IQR $697–$9459) $8637 (mean, SD not reported); $2726 (median, IQR $385–$8237) 2025 USD
Dismuke-Greer et al., 2020	Four large Veterans Affairs Medical Centers	2002–2016	Healthcare costs. Annual outpatient healthcare costs during entire study period.	Cost data from VA facilities and Medicare estimates using the relative values of CPT codes	$4901 (mean, 95% CI $4392–$5468), non-blast-related mTBI, N = 193 $6480 (mean, 95% CI $5842–$7187), blast-related mTBI, N = 207 $5721 (weighted mean, 95% CI $5284–$6152), N = 400 2018 USD	$6282 (mean, 95% CI $5629–$7008), non-blast-related mTBI, N = 193 $8305 (mean, 95% CI $7487–$9211), blast-related mTBI, N = 207 $7332 (weighted mean, 95% CI $6853–$7979), N = 400 2025 USD
Cogan et al., 2022	IQVIA Integrated Data Warehouse	2011–2016	Healthcare costs. Included pharmacy, outpatient, and inpatient visits during 24 mo post-injury.	IQVIA Integrated Data Warehouse	$45,680 (mean, SD $72,630), mTBI + CVI, N = 20,441 $28,563 (mean, SD $48,443), mTBI only, N = 20,441 $37,121.50 (weighted mean, SD $62,322), N = 40,882 2017 USD	$59,757 (mean, SD $95,011), mTBI + CVI, N = 20,441 $37,365 (mean, SD $63,371), mTBI only, N = 20,441 $48,561 (weighted mean, SD $82,501), N = 40,882 2025 USD
Dalton et al., 2022	Military Health System Data Repository	2007–2011	Healthcare costs. Included encounters at military facilities, civilian facilities, and pharmacies. Annual expenditure during each subsequent yr after the first yr post-injury.	Military Health System Data Repository	$11,270 (median, IQR $3977–$24,342) 2019 USD	$14,223 (median, IQR $5019–$30,721) 2025 USD
Richard et al., 2025	Military Data Repository	2011–2021	Healthcare costs. Average cost of a primary care visits for the patient with a provider over the study period.	Costs based on relative value units, adjusted by the Ambulatory Payment Classification weight and multiplied by the Centers for Medicare and Medicaid Services conversion factor to determine the total cost in military treatment facilities	$538.9 (mean, 95% CI $525.14–$552.24), military members, N = 44,568 $519.3 (mean, 95% CI $507.94–$530.27), civilians, N = 6441 $536.40 (weighted mean, 95% CI $524.30–$548.14), N = 51,009 2021 USD	$654 (mean, 95% CI $638–$671), military members, N = 44,568 $631 (mean, 95% CI $617–$644), civilians, N = 6441 $651 (weighted mean, 95% CI $637–$666), N = 51,009 2025 USD

**Caption:** This table summarizes all included studies reporting healthcare costs incurred beyond the first year following mild TBI. Extracted variables include study setting, study years, description of the cost or charge categories, method of cost or charge estimation, original reported values, and corresponding 2025 USD equivalents following inflation adjustment.

Therefore, the reference year for the reported USD values was assumed to correspond to the final year of the study period.

CPT, Current Procedural Terminology; CVI, chronic vestibular impairment; IQR, interquartile range; CI, confidence interval; mTBI, mild traumatic brain injury; mo, months; N, sample size; SD, standard deviation; TBI, traumatic brain injury; USD, United States dollars; VA, Veterans Affairs; yr/yrs, year/years.

a The study did not specify the reference year for reported costs or charges.

**Fig. 4:**
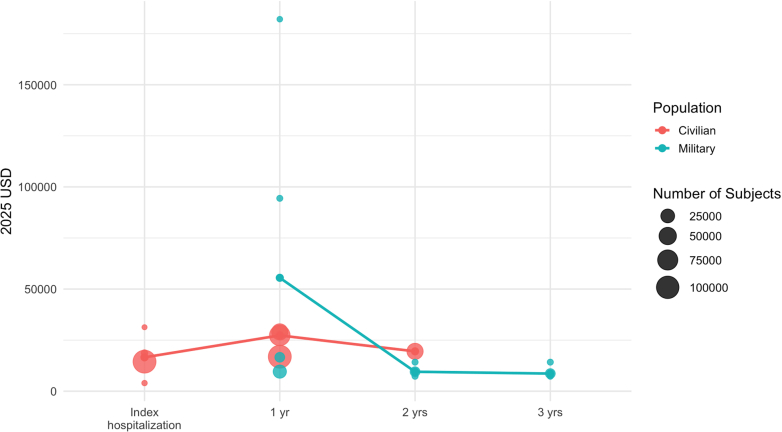
Temporal trends in healthcare costs among civilian and military populations for mild traumatic brain injury. This figure illustrates reported healthcare costs at the index hospitalization and at 1-, 2-, and 3-year follow-up intervals for civilian and military cohorts. Each point represents an individual study population, with point size proportional to sample size. Lines connect median costs for civilian and military groups. All values are standardized to 2025 U.S. dollars. TBI, traumatic brain injury; USD, United States dollars.

Trends in healthcare costs over time among civilian and military populations are shown in Fig. 4. During the first-year post-injury, military cohorts demonstrated a substantially wider range of costs compared with civilians, with the highest military estimates exceeding any civilian estimates. By the second year post-injury, cost ranges for military and civilian populations converged. Among civilians, second-year expenditures approximated those incurred during the index hospitalization.

## Discussion

This scoping review synthesizes three decades of U.S. data on the direct medical costs of mTBI across acute, post-acute, and long-term phases of care. Although definitions and analytic frameworks varied, the collective evidence demonstrates that mTBI imposes a substantial and persistent economic burden extending well beyond the initial hospitalization. This points to the need for a balanced approach to ensuring continued follow-up implementing patient specific, efficacious interventions with healthcare delivery and reimbursement models that enable efficient and cost-effective treatment of patients with mTBI.

There is a widening divergence between reported healthcare costs and charges for acute mTBI care. While average per-admission costs for mTBI hospitalizations in national datasets remained broadly stable over time, healthcare charges rose dramatically over the same period, with single-center contemporary reports as high as $172,080 per mTBI encounter. This cost-to-charge gap is not unique to mTBI; national cost-to-charge ratios have fallen from about 0.70 in the mid-1990s to roughly 0.32 in 2020. This divergence likely reflects the increasing complexity of hospital charge structures, Medicare reimbursement methods, and shifts in negotiated payer contracts.^26^^,^^27^ Rising charges have important implications for trauma-system sustainability, and may reflect hospital efforts to offset reimbursement that lags behind actual care costs or hospital market power. Urban safety-net hospitals, where neurotrauma is common and Medicaid reimbursement remains far below commercial rates, may be particularly vulnerable to financial strains.^28^^,^^29^ It is therefore critical to ensure reimbursement models for centers that treat these patients to reflect true resource demands.

Beyond the index hospitalization, this review shows that post-acute and chronic care are major contributors to total mTBI-related spending. Within the first year after injury, direct healthcare costs for patients engaged in intensive, structured neurorehabilitation programs within VA and DoD systems ($182,094) were higher than those reported in large civilian claims datasets ($16,898–$29,045). These expenditures reflect not only traditional medical encounters (follow-up clinic visits, neuroimaging, prescription management) but also prolonged rehabilitation services. In veteran and active-duty populations, multidisciplinary post-concussive care is often coordinated within vertically integrated systems that bundle physical rehabilitation, cognitive retraining, behavioral health, and vocational support.^30^^,^^31^ It is possible that high early expenditures and front-loading of rehabilitative resources may optimize long-term functional recovery and reduce long-term disability costs.^32^^,^^33^

In contrast, the civilian care pathway for mTBI is comparatively fragmented. Many patients diagnosed with a concussion or mild head injury are discharged from the emergency department, and subsequent care is dispersed across outpatient primary care, urgent care, and physical therapy.^34^, ^35^, ^36^ This diffusion makes the true cumulative cost of care difficult to capture in any single administrative source. It also creates the risk that under-resourced patients—those without robust commercial coverage or access to mTBI care—incur avoidable downstream costs through repeated emergency utilization, duplicative imaging, or prolonged disability.^37^^,^^38^ The demonstrated divergence between civilian and military/VA cost trajectories further suggests that the front-loaded, integrated rehabilitation model employed in VA and DoD systems may provide a scalable template for civilian value-based care reform, though prospective comparative effectiveness data are needed for confirmation. There remain critical issues including access to care, healthcare payment models, and lack of uniformity in healthcare delivery across institutions and regions, which make care reform challenging. A clearer understanding of head injury-related costs will aid in informing healthcare policy and discussions around disease burden and resource allocation.

Importantly, VA or DoD studies often use managerial cost accounting to estimate resource utilization, whereas some civilian studies rely on billed charges or claims data converted using cost-to-charge ratios.^39^ These differences in cost-accounting methodology further limit direct comparability across systems. Moreover, access to and intensity of post-acute rehabilitation may vary across insurance plans, potentially influencing both utilization patterns and downstream costs. Differences in rehabilitation benefits may therefore contribute to heterogeneity in reported post-acute expenditures.

Those who sustain mTBI are at increased risk of chronic multisystem disease—including cardiometabolic, neuropsychiatric, and other medical conditions—that may emerge or worsen years after the index injury and are not fully captured in conventional cost analyses.^40^^,^^41^ Importantly, the term “mild traumatic brain injury” is an imprecise descriptor of a heterogeneous condition.^42^ The economic burden following injuries currently classified as mTBI may vary substantially based on pre-injury risk factors (e.g., mental health conditions, socioeconomic disadvantage, and prior TBI), injury complexity, and access to post-acute rehabilitation.^43^, ^44^, ^45^ The 2025 NINDS CBI-M Framework reflects a field-wide shift towards multidimensional TBI characterization and individualized patient- and injury-specific TBI classification.^7^ As this framework becomes adopted, it may enable more accurate attribution of healthcare charges and costs to specific injury profiles and recovery trajectories.

Taken together, these findings point to two policy-relevant conclusions. First, mTBI should not be viewed as a brief, self-limited encounter with minimal financial impact. Patients frequently generate substantial utilization over months to years from emergency department, primary care, and specialty care visits. This pattern aligns with the known clinical trajectory of post-concussive symptoms—headache, vestibular dysfunction, cognitive slowing, sleep disturbance, mood changes—which may persist and impair function long after the initial event.^46^^,^^47^ Second, the distribution of costs over time suggests that economic value in mTBI care may depend less on marginal reductions in the initial emergency department or inpatient episode and more on preventing chronic, symptom-driven disability. As we consider nationally how to best fund and deliver healthcare, the rising charges associated with mTBI and long-term costs point to the burden of mTBI not just as an isolated, inconsequential part of healthcare economics, but rather as a complex and long-lasting disease that requires thoughtful and innovative changes in healthcare.

Our findings provide payers and health systems with the actuarial basis to justify upfront investment in early, structured rehabilitation. There is growing evidence that targeted, protocolized rehabilitation—including vestibular therapy, graded aerobic exercise, and structured return-to-work programs—can accelerate symptom resolution.^48^^,^^49^ Furthermore, multidisciplinary rehabilitation programs may shorten recovery and improve return-to-work outcomes compared with traditional approaches.^50^^,^^51^ However, despite growing evidence for clinical benefit, the cost-effectiveness and long-term economic impact of these interventions remain incompletely defined. From a health systems standpoint, identifying subgroups who continue to incur high costs after the first year would allow targeted deployment of follow-up resources rather than low-yield surveillance of everyone with a mild head injury. Future work should focus on prospective, longitudinal studies that integrate both cost and patient-centered outcomes to identify high-value interventions.

In sum, mTBI is far from economically benign. The financial footprint is diffuse, long-lived, payer-dependent, and tightly linked to functional recovery and access to coordinated rehabilitation. Better economic data—especially prospective, civilian, longitudinal data that integrate utilization, functional status, and work participation—are required to design value-based care pathways that prioritize recovery rather than reactively and repeatedly treating persistent symptoms.

This review has several limitations. First, the included studies demonstrated substantial heterogeneity in patient populations, data sources, and cost-accounting methodologies. Cohorts spanned both civilian and military settings, and data sources ranged from single-center trauma registries to large national claims databases. Variability in mTBI definitions likely contributed to inconsistency in case ascertainment, and payer structures and reporting practices differed across institutions. In addition, the specific cost components included—such as inpatient, outpatient, pharmacy, rehabilitation, or ancillary services—varied across studies, further limiting uniformity and comparisons across studies. Insurance plan–level variation in rehabilitation benefits was not reported in included studies and could not be controlled for in this review. Variability in adherence to CHEERS reporting domains introduces potential bias in cost estimation. Specifically, moderate quality studies often only had costs related to the index hospitalization, while high quality studies often allowed for more comprehensive longitudinal analyses. Collectively, these factors constrain direct cost comparisons and the ability to synthesize a unified estimate of mTBI-associated expenditures.

Second, our investigation specifically evaluated direct medical costs associated with mTBI. It did not account for indirect or societal costs, such as productivity loss, caregiver burden, or reduced quality of life. As a result, the true economic burden of mild traumatic brain injury is likely substantially underestimated. For example, in looking at employment and economic outcomes for patients with mTBI from large multicenter U.S. data, 17% of patients previously employed were not working at 12 months after their injury.^38^

Additionally, this review searched a single database, which may have resulted in failure to identify eligible studies indexed in other repositories. These findings are specific to the U.S. healthcare context; differences in healthcare financing, delivery systems, and insurance structures limit direct applicability to other national health systems. Furthermore, the heterogeneity of included studies led to the presentation of each case on a study-by-study basis within each phase-of-care section. Future studies employing more standardized cost-accounting methodologies and consistent mTBI definitions would facilitate more formal evidence synthesis.

Finally, several studies lacked granular data on cost components (e.g., inpatient versus outpatient services, rehabilitation, pharmacy), limiting detailed phase-specific comparisons. Mechanism of injury was inconsistently reported and not stratified in cost analyses across included studies. Differences in mechanism—such as blast-related and combat-associated exposures in military populations—may influence comorbidity burden and downstream healthcare utilization, contributing to cost heterogeneity and limiting comparability. Additionally, race/ethnicity and sex/gender were not routinely reported, precluding analysis of demographic variation in mTBI costs. Despite these limitations, the synthesis provides the most comprehensive summary to date of direct healthcare costs associated with mTBI across the continuum of care.

The economic burden of mild traumatic brain injury (mTBI) extends well beyond the initial encounter, encompassing substantial post-acute and long-term healthcare utilization across diverse care settings. Although mTBI is associated with low mortality and short hospital stays, expenditures accrue through outpatient, rehabilitation, and mental health services related to persistent functional symptoms. This diffuse, longitudinal burden is distinct from that incurred by conditions characterized by procedural intensity or high acute mortality. The costs and long-term health consequences of mTBI remain under-appreciated in examinations of mTBI as a chronic disease course with both acute and longitudinal disability, in comparison with other well-known diseases such as cancer, stroke, or heart disease, where the long-term burden is better acknowledged. The widening gap between healthcare costs and rising charges underscores the need for reimbursement models that more accurately reflect the true resource demands of mTBI care. Standardized cost-reporting frameworks and coordinated, value-based models of acute and long-term care will be essential to improving resource allocation and reducing the overall societal impact of mTBI.

## Contributors

JKY, DJC, and KRR conceived and designed the study. JKY and DJC provided supervision to KRR. KRR conducted the database searches, study screening, and data preparation. DJC and KRR resolved conflicts regarding study inclusion. JKY, DJC, and KRR contributed to data interpretation, drafting of the manuscript, and figure preparation. PET, CH, AAA, MCVV, RS, AMD, MCH, HEH, and GTM contributed to data interpretation and critically revised the manuscript. All authors reviewed the study findings, critically revised the manuscript, and approved the final version before submission. JKY, DJC, and KRR accessed and verified the data. JKY reviewed all materials and made the final decision to submit on behalf of the authors.

## Data sharing statement

All data reported and analyzed in this scoping review were derived from published reports available from the National Library of Medicine PubMed database. Data contained within this review can be made available to researchers upon reasonable request to the corresponding author at John.Yue@ucsf.edu. Please provide the rationale for data access and its proposed use when submitting a data request. Data related to this study can be provided with investigator support.

## Declaration of interests

The authors declare the following funding sources, which were not applicable to the work contained within this scoping review: National Institute of Neurological Disorders and Stroke, National Institutes of Health, through UCSF grant number 2UE5NS070680-17 (to David J. Caldwell); Pac-12 Student Health and Wellbeing Initiative, the Weill Institute for Neurosciences Neurohub, the UC Noyce Initiative, the National Institute on Deafness and Other Communication Disorders, and the NIH StrokeNet (to Cathra Halabi); grants from DePuy Synthes, Florida Essential Healthcare Partnership, and the Charles Koch Foundation (to Anthony M. DiGiorgio); National Institute of Neurological Disorders and Stroke, National Institutes of Health, #1K23NS110828 (to H. E. Hinson); National Institute of Neurological Disorders and Stroke, National Institutes of Health, #RC2NS069409, #U01NS086090, #U01NS1365885, United States Department of Defense #W81XWH-13-1-0441, #W81XWH-14-2-0176, #W81XWH-18-2-0042 (to Geoffrey T. Manley); UCSF Weill Neurohub Clinician-Scientist Award (Project ID #7032139, 2025–2026), UCSF Weill Institute for the Neurosciences and UCSF Innovation Ventures Catalyst Award Grant (UCSF Award #7032446, 2025–2026), Congress of Neurological Surgeons Foundation Sophysa Young Investigator Award (UCSF Proposal ID #P0594482, 2026–2027) (to John K. Yue).

RS reports personal fees from Proprio, Spiderwort, and Globus, outside the submitted work. CH reports unpaid roles with the National Academies of Sciences, Engineering, and Medicine Action Collaborative on Traumatic Brain Injury and the NIH StrokeNet Recovery and Rehabilitation working groups. All other authors declare no competing interests.
