# Supplementary material for: Economic burden of adult mild traumatic brain injury in the United States: a scoping review of healthcare-related charges and costs
Source: Lancet Reg Health Am. 2026 Jul 6;62:101558. doi: 10.1016/j.lana.2026.101558 (PMC13355770; doi:10.1016/j.lana.2026.101558)
Supplement: Supplementary Material [file mmc1.pdf]

Supplementary Online Content

Ran KR, Caldwell DJ, Tarapore PE, et al. Title: Economic Burden of Adult Mild Traumatic Brain Injury in the United States. Subtitle: A Scoping Review of Healthcare-Related Charges and Costs. *Lancet Regional Health Americas*

Supplementary Table 1.
Search strategy for studies evaluating healthcare costs and charges associated with mild TBI

Supplementary Table 2.
Geographic distribution of civilian and military study populations across United States regions

Supplementary Table 3.
Newcastle-Ottawa Scale quality assessment of included studies

Supplementary Table 4.
CHEERS-based quality assessment of included studies

Supplementary Table 1. Search strategy for studies evaluating healthcare costs and charges associated with mild TBI

Search Concept	Field	Search Terms and Strategy	Notes and Filters
Traumatic Brain Injury	Title/Abstract	(brain injury) OR (brain injuries) OR (brain trauma) OR (head injury) OR (head injuries) OR (head trauma) OR (intracranial injury) OR (intracranial injuries) OR (intracranial trauma) OR (cerebral injury) OR (cerebral injuries) OR (cerebral trauma) OR (cranial trauma) OR (cranial injury) OR (cranial injuries) OR (craniocerebral trauma) OR (craniocerebral injury) OR (craniocerebral injuries) OR (concussion) OR (concussive) OR (post-concussion) OR (post-concussive)	Captures all synonyms and variations of traumatic brain injury-related terminology.
Costs / Economic Terms	Title/Abstract	(cost) OR (costs) OR (expenditure) OR (expenditures) OR (expense) OR (expenses) OR (claim) OR (claims) OR (charge) OR (charges) OR (payment) OR (payments) OR (economic) OR (economics) OR (financial) OR (money) OR (monetary) OR (fiscal) OR (utilization) OR (cost-effectiveness) OR (cost-utility) OR (cost-benefit) OR (cost-analysis) OR (income) OR (consumption)	Captures financial, economic, and resource utilization terms.
Severity / Classification	All Fields	(GCS) OR (Glasgow Coma) OR (AIS) OR (Abbreviated Injury Scale) OR (Abbreviated Injury Score) OR (CT) OR (computed tomography) OR (MRI) OR (imaging) OR (mild) OR (minimal) OR (moderate) OR (severe)	Includes injury grading systems, imaging, and severity descriptors.
Exclusion: Pediatrics	Title/Abstract	(pediatric) OR (adolescent) OR (adolescents) OR (neonate) OR (neonates) OR (infant) OR (infants) OR (children)	Excludes pediatric populations.
Exclusion: Animals	Title/Abstract	(animal) OR (rat) OR (rats) OR (mouse) OR (mice) OR (murine) OR (pig) OR (swine) OR (porcine)	Excludes animal studies.
Results	—	—	Yields 3,302 studies (as of October 2025).

Caption: PubMed search strategy employed by this scoping review to identify studies reporting healthcare costs or charges related to mild TBI, using a combination of Medical Subject Headings (MESH) terms and keywords. AIS = Abbreviated Injury Scale; CT = computed tomography; GCS = Glasgow Coma Scale; MRI = magnetic resonance imaging; TBI = traumatic brain injury

Supplementary Table 2. Geographic distribution of civilian and military study populations across United States regions.

Population	Region	Setting	State	N	Study
Civilian (N=6,151)	Northeast (N=846, 13.8%)	Level I/II trauma center	NY	701	Ranson et al. 2024
		Level I/II trauma center	NH	145	Root et al. 2020
	Midwest (N=640, 10.4%)	Level I/II trauma center	ND	44	Beard et al. 2025
		Level I/II trauma center	OH	596	Harris et al. 2024
	South (N=4,535, 73.7%)	Level I/II trauma center	TX	2855	Salisbury et al. 2017
		Level I/II trauma center	TX	1447	Dengler et al. 2020
		Level I/II trauma center	VA	143	Martyak et al. 2018
		Level I/II trauma center	WV	90	Dobbs et al. 2025
	West (N=130, 2.1%)	Level I/II trauma center	CA	130	Shen et al. 2024
Military (N=649)	Midwest (N=29, 4.5%)	Military/VA system	MN	29	Dismuke-Greer et al. 2023
	South (N=443, 68.3%)	Military/VA system	DC	178	Richardson et al. 2018
		Military/VA system	FL	29	Dismuke-Greer et al. 2023
		Military/VA system	NC	178	Richardson et al. 2018
		Military/VA system	TX	29	Dismuke-Greer et al. 2023
		Military/VA system	VA	29	Dismuke-Greer et al. 2023
	West (N=177, 27.3%)	Military/VA system	CA	29	Dismuke-Greer et al. 2023
		Military/VA system	CA	148	Darr et al. 2025

Caption: This table summarizes the geographic distribution of included civilian (N = 6,151) and military (N = 649) study populations among 17 studies with identifiable sites, excluding studies that used national databases without site-level geographic information. Civilian and military cohorts were subdivided by United States Census region. Regional percentages were calculated relative to the total civilian or military study population, respectively. For studies by Richardson et al. (2018) and Dismuke-Greer et al. (2023), site-specific population sizes were not reported and therefore total cohort sizes were evenly divided across listed sites to estimate the contribution of each region. CA = California; DC = Washington, District of Columbia; FL = Florida; MN = Minnesota; NC = North Carolina; ND = North Dakota; NH = New Hampshire; NY = New York; OH = Ohio; TX = Texas; VA = Veterans Affairs; VA = Virginia; WV = West Virginia

Supplementary Table 3. Newcastle-Ottawa Scale quality assessment of included studies

	Selection				Comparability		Outcome			NOS Total */9	NOS Quality
**Publication**	S1	S2	S3	S4	C1	C2	O1	O2	O3		
Schootman et al. 2003	*	-	*	-	*	*	*	*	*	7/9	High
Farhad et al. 2013	*	-	*	-	*	*	*	*	*	7/9	High
Salisbury et al. 2017	*	-	*	*	*	-	*	*	*	7/9	High
Taylor et al. 2017	*	*	*	*	*	*	*	*	*	9/9	High
Martyak et al. 2018	*	*	*	*	*	-	*	*	*	8/9	High
Richardson et al. 2018	*	*	*	*	*	*	*	*	*	9/9	High
Pavlov et al. 2019	*	-	*	*	-	-	*	*	*	6/9	Moderate
Dengler et al. 2020	*	*	*	*	*	*	*	*	*	9/9	High
Dismuke-Greer et al. 2020	*	*	*	*	*	*	*	*	*	9/9	High
Root et al. 2020	*	*	*	*	*	-	*	*	*	8/9	High
Cogan et al. 2022	*	*	*	*	*	*	*	*	*	9/9	High
Dalton et al. 2022	*	*	*	*	*	*	*	*	*	9/9	High
Dismuke-Greer et al. 2023	*	*	*	*	*	*	*	*	*	9/9	High
Harris et al. 2024	*	-	*	*	*	-	*	*	*	7/9	High
Ranson et al. 2024	*	-	*	*	*	*	*	*	*	8/9	High
Shen et al. 2024	*	-	*	*	*	-	*	*	*	7/9	High
Beard et al. 2025	*	*	*	*	*	-	*	*	*	8/9	High
Darr et al. 2025	*	*	*	*	*	*	*	*	*	9/9	High
Dobbs et al. 2025	*	-	*	*	*	-	*	*	*	7/9	High
Marcet et al. 2025	*	-	*	*	*	-	*	*	*	7/9	High
Richard et al. 2025	*	*	*	*	*	*	*	*	*	9/9	High

Caption: NOS = Newcastle-Ottawa Scale. Selection: S1 = representativeness of exposed cohort; S2 = selection of non-exposed cohort; S3 = ascertainment of exposure; S4 = outcome not present at start of study. Comparability: C1 = comparability on main factor; C2 = comparability on additional factor (maximum 2 stars). Outcome: O1 = assessment of outcome; O2 = adequacy of follow-up length; O3 = adequacy of follow-up. * = 1 star awarded; ** = 2 stars awarded; - = not awarded. High quality = 7–9 stars; Moderate quality = 4–6 stars; Low quality = <4 stars.

Supplementary Table 4. CHEERS-based quality assessment of included studies

Publication	Title and Abstract	Background and Objectives	Study Population (mTBI Defined)	Setting and Location	Time Horizon	Cost Type	Costing Method	Price Year and Currency	Characterizing Heterogeneity	Characterizing Uncertainty	Overall Quality	Quality Rating Scheme for Studies and Other Evidence (1-5)
Schootman et al. 2003	Yes	Yes	Yes	Yes	Yes	Yes	Yes	No	Yes	Yes	Moderate	3
Farhad et al. 2013	Yes	Yes	Yes	Yes	Yes	Yes	Yes	No	Yes	Yes	Moderate	3
Salisbury et al. 2017	Yes	Yes	Yes	Yes	Yes	Yes	Yes	No	Yes	Yes	Moderate	3
Taylor et al. 2017	Yes	Yes	Yes	Yes	Yes	Yes	Yes	No	Yes	Yes	Moderate	3
Martyak et al. 2018	Yes	Yes	Yes	Yes	Yes	Yes	Yes	No	Yes	No	Moderate	3
Richardson et al. 2018	Yes	Yes	Yes	Yes	Partial	Yes	Yes	No	Yes	Yes	Moderate	3
Pavlov et al. 2019	Yes	Yes	Yes	Yes	Yes	Yes	Yes	Yes	Yes	Yes	High	3
Dengler et al. 2020	Yes	Yes	Yes	Yes	Yes	Yes	Yes	No	Yes	Yes	Moderate	3
Dismuke-Greer et al. 2020	Yes	Yes	Yes	Yes	Yes	Yes	Yes	Yes	Yes	Yes	High	3
Root et al. 2020	Yes	Yes	Yes	Yes	Yes	Yes	Yes	No	Yes	Yes	Moderate	3
Cogan et al. 2022	Yes	Yes	Yes	Yes	Yes	Yes	Yes	Yes	Yes	Yes	High	3
Dalton et al. 2022	Yes	Yes	Yes	Yes	Yes	Yes	Yes	Yes	Yes	Yes	High	3
Dismuke-Greer et al. 2023	Yes	Yes	Yes	Yes	Yes	Yes	Yes	Yes	Yes	Yes	High	3
Harris et al. 2024	Yes	Yes	Yes	Yes	Yes	Yes	Yes	No	Yes	No	Moderate	3
Ranson et al. 2024	Yes	Yes	Yes	Yes	Yes	Yes	Yes	No	Yes	Yes	Moderate	3

Shen et al. 2024	Yes	Yes	Yes	Yes	Yes	Yes	Yes	No	Yes	No	Moderate	3
Beard et al. 2025	Yes	Yes	Yes	Yes	Yes	Yes	Yes	No	Yes	No	Moderate	3
Darr et al. 2025	Yes	Yes	Yes	Yes	No	Yes	Yes	Yes	Yes	No	Moderate	2
Dobbs et al. 2025	Yes	Yes	Yes	Yes	Yes	Yes	Yes	No	Yes	No	Moderate	3
Marcet et al. 2025	Yes	Yes	Yes	Yes	Yes	Yes	Yes	Yes	Yes	Yes	High	3
Richard et al. 2025	Yes	Yes	Partial	Yes	Yes	Yes	Yes	Yes	Yes	Yes	Moderate	3

Caption: CHEERS = Consolidated Health Economic Evaluation Reporting Standards; mTBI = mild traumatic brain injury
